# Gallbladder cancer during pregnancy treated with surgery and adjuvant gemcitabine: A case report and review of the literature

**DOI:** 10.3389/fonc.2022.1006387

**Published:** 2022-10-24

**Authors:** A. Diciolla, M. Gianoni, M. Fleury, P. Szturz, N. Demartines, S. Peters, R. Duran, D. Desseauve, Monnat A. Panchaud, F. Fasquelle, A. Digklia

**Affiliations:** ^1^ Département d’Oncologie, Centre Hospitalier Universitaire Vaudois (CHUV), Lausanne, Switzerland; ^2^ Faculty of Biology and Medicine, University of Lausanne, Lausanne, Switzerland; ^3^ University of Lausanne (UNIL) et Service de Gynécologie, CHUV, Lausanne, Switzerland; ^4^ Department of Visceral Surgery, CHUV, Lausanne, Switzerland; ^5^ Department of Radiology and Interventional Radiology, Lausanne University Hospital, Lausanne, Switzerland; ^6^ Women-Mother-Child Department, Centre Hospitalier Universitaire Vaudois, Lausanne, Switzerland; ^7^ Service of Pharmacy Service, Lausanne University Hospital and University of Lausanne, Lausanne, Switzerland; ^8^ Institut Universitaire de Pathologie, Pathologie Clinique, Centre Hospitalier Universitaire Vaudois (CHUV), Lausanne, Switzerland

**Keywords:** gallbladder cancer, chemotherapy, pregnancy, gemcitabin, biliary tract cancer

## Abstract

**Background:**

Gallbladder cancer (GBC) represents the most common biliary tract cancer. Prognosis remains poor with 5-year overall survival rates less than 5% in advanced stages. GBCs are diagnosed more frequently in women, supposedly due to endocrine factors.

**Case:**

A 35-year-old woman, diagnosed with a non-metastatic GBC in the 22nd week of gestation, underwent a complete surgical resection 5 weeks later. Adjuvant gemcitabine was administered without complications, temporarily discontinued in the 32nd week to allow childbirth. The patient was disease-free for more than 3 years with ongoing remission at the last visit in July 2022. During the follow-up period, the child had no developmental, cognitive, or other health issues.

**Conclusion:**

Malignant tumors occur in about 0.1% of pregnant women, many are treated with chemotherapy. In oncology, the need to deliver optimal treatment in these patients represents a major concern. Both surgery and adjuvant chemotherapy of locally advanced GBC can be performed safely, with certain considerations, in the second trimester of pregnancy.

## Introduction

Gallbladder cancer (GBC) is considered a highly fatal hepatobiliary malignancy due to its poor 5- year survival rate of 0-12% and median overall survival (OS) of less than 6 months in untreated patients (1). Worldwide, there is prominent geographic variability in GBC incidence (2), which can be illustrated by its distribution on the American continent, with high rates seen in South American countries (for example incidence ≥1.6 per 100,000 individuals in Chile) strongly contrasting with the US 0.56-0.82 low-incidence. While socio-economic factors explain these disparities (i.e. delayed access to cholecystectomy for gallstones (3)) the strong gender difference in the distribution of the disease does not. Even in low-incidence countries, women are affected 2-3 times more frequently than men (2). The association of GBC with the female gender has been hypothesized to be influenced by estrogens leading to an increase in the supersaturation of bile cholesterol, being therefore potentially involved in the pathogenesis of GBC mediated by gallstones (4). This over-representation of female gender, unfortunately, increases the risk for diagnoses during pregnancy. In the absence of metastasis, surgery and adjuvant chemotherapy are the cornerstones of treatment, but exposure during pregnancy to molecules described as deleterious to the fetus (5, 6) may lead to a decision to terminate the pregnancy or forego treatment leading to dramatic consequences (7). In order to provide data for this critical decision making, the case presented here is a pregnant patient diagnosed with GBC and treated by surgery and adjuvant gemcitabine chemotherapy during pregnancy with a follow-up of more than 3 years.

## Case description

**Figure 1:**
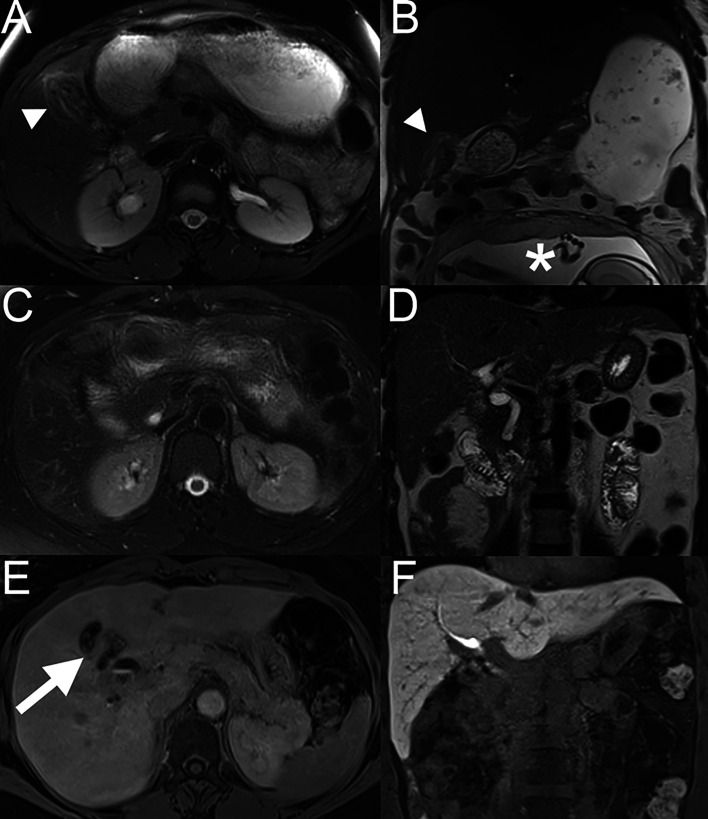
Liver MRI done 2 weeks after cholecystectomy. On axial fat-suppressed T2-weighted TSE **(A)** and coronal T2-weighted **(B)** images, mild hyperintense signal is seen at the edge of the surgical resection in segment IV and V. (*) Pregnant uterus at 26 weeks of amenorrhea. Liver MRI at 7 months follow-up, on axial fat-suppressed T2-weighted TSE **(C)** and coronal T2- weighted **(D)** images, the signal hyperintensity previously seen has disappeared. On the hepatobiliary phase (axial **(E)** and coronal **(F)** view), acquired at 20 minutes after injection of gadoxetic acid (Primovist), no metastases were seen. Some metal artefacts related to surgical clips post-cholecystectomy can been seen (arrow; **E**).

In February 2019, a 35-year-old African woman in the 22nd week of gestation presented with acute biliary pancreatitis due to gallbladder polyps. Two weeks later, the patient underwent a cholecystectomy by laparoscopy without maternal nor fetal complications. The histopathological examination revealed an incidental gallbladder carcinoma classified as pT2a pNx L0 V0 G2 R0 according to the American Joint Committee on Cancer staging system (8th edition). Additional examinations were therefore carried out, including a non-contrast magnetic resonance imaging (MRI) two weeks after the first surgery (**Figure 1**). Serum tumor markers including carbohydrate antigen 19-9 (CA 19-9) and carcinoembryonic antigen (CEA) were within normal limits (< 2 kU/L and 1.8 µg/L, respectively) and the expression of MMR proteins (MLH1, MSH2, MSH2 and PMS2) and HER-2 by immunohistochemistry in tumor tissue were normal. After a discussion with the patient, particular care was dedicated to maintain the pregnancy that the patient wished to preserve. Following multidisciplinary tumor board discussion, the patient underwent an open liver bi-segmentectomy of segments IVb and V showing two retro-portal lymph node metastases of GBC with one of them presenting capsular effraction. The tumor was classified pT2a pN1(2/8), grade 2, without lymphovascular invasion, stage IIIB. No postoperative complications were observed. Fetal growth, liquor volume, and end-diastolic flow on ultrasound at 26 weeks were normal.

After an explanation about the benefit-risk balance of adding chemotherapy treatment, the patient began adjuvant gemcitabin and the pregnancy was carried on until 32 weeks of gestation. Because of the risk of preterm delivery, the patient received corticosteroids for fetal lung maturation before hepatic surgery.

Between the 28th and 32nd week of pregnancy, the patient was treated with one cycle of gemcitabine chemotherapy (1000 mg/m2 on days 1 and 8 of a 21-day cycle) with no grade 2 or more adverse events. Chemotherapy was administered on an inpatient basis to facilitate fetal monitoring, and no adverse fetal effects were observed. The treatment was interrupted 2 weeks prior to the planned birth induction at 32 weeks. Because of breech position at admission, the patient underwent a cesarean section. The newborn girl had a birth weight of 1,570 g, a length of 41 cm, and a head circumference of 28.5 cm (all values in the normal range for gestational age). The Apgar score was 7/9/9 at 1, 5 and 10 minutes respectively.The placenta showed no signs of metastatic spread. A complete neonatal evaluation revealed no gross malformations, neurological or cardio-circulatory abnormalities, infection, or bone marrow suppression. Peripheral blood count was as follows: leukocytes 16.5x109/L, hemoglobin 17.3 g/dL, and platelets 417,000/µL). Supplemental enteral nutrition by nasogastric tube was required for the first 6 days. The infant developed indirect hyperbilirubinemia with jaundice and hemoglobin level of 13.2 g/dl on day 14, requiring iron infusion.

The infant was discharged from the hospital in very good general condition one month after the birth. Her weight was 2,380 g (below the third percentile), height 45 cm (below the third percentile), and head circumference 31 cm (below the third percentile). Pediatric follow-up at 8 and 18 months of corrected age confirmed normal physical and neurological development. At the latter visit, the infant’s weight was 13 kg (above the 97th percentile), her height 87 cm (above the 97th percentile), and head circumference 48.5 cm (between the 75th and 90th percentile).

At 6 weeks postpartum, chemotherapy with gemcitabine was resumed using the same dosing schedule as during the pregnancy, in combination with capecitabine at 1500 mg orally twice a day for the first two weeks of every 3-week cycle and a total of 6 months were administered.

**Figure 2:**
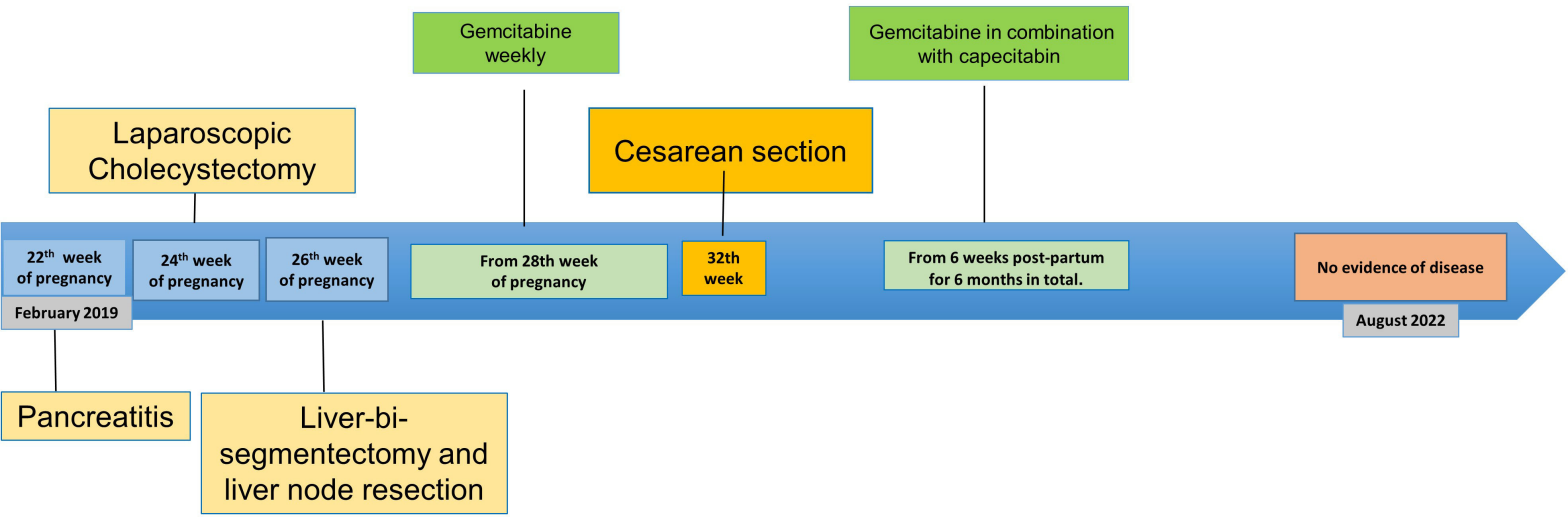
Timeline of disease course.

After finishing chemotherapy, the first follow-up imaging by abdominal MRI and thoraco- abdominal computed tomography (CT) was performed in October 2019, revealing no signs of local recurrence or distant metastases (**Figures 1C–F**). Further oncologic surveillance included clinical, laboratory (CEA, CA 19-9) and radiological monitoring. In this respect, the patient had a lung CT and abdominal MRI every three months in the first two years after the intervention, then the intervals were increased to every six months. This patient has been disease-free for more than 3 years with ongoing remission at the last visit in February 2022. **Figure 2** summarizes the disease course in our patient.

## Discussion

There is an increasing number of cancers discovered during pregnancies, as shown by several contemporary epidemiological studies (8). Concerning surgery, it can be safely carried out at any time during pregnancy. However, a higher risk of miscarriage has been reported in the first trimester. Also, the risk of small for gestational age fetuses has been reported when a pregnant patient undergoes a long abdominal procedure during pregnancy. Moreover, major abdominal and pelvic surgery is associated with increased morbidity and pregnancy complications, like premature delivery, throughout the whole gestation period. Therefore, watchful waiting policy can also be considered. Nevertheless, when the patient is at high risk for torsion (usually at gestational weeks 8-16), rupture infarction, or acute abdomen, surgical management is indicated (9).

If a choice is possible, the second trimester is preferable for surgery. Nevertheless, surgery should never be postponed if deemed to be crucial for patient care management (10).

Given the propensity of GBC to spread to the regional lymph nodes and peritoneal surface, staging laparoscopy can help avoid unnecessary laparotomy in 38-62% of patients. Based on the aggressivity of the disease, staging laparoscopy is recommended in all cases of suspected or proven GBC. As a result, only 25% of patients will undergo potentially curative surgery and only 16% will survive more than 5 years (10). After curative surgery for biliary tract cancers, the ideal adjuvant chemotherapy has not been established but most centers used adjuvant capecitabine for 6 months based on the BILCAP phase III study. In this trial, although no significant difference in OS was observed, in an unadjusted intent-to-treat (ITT) analysis, a per-protocol analysis revealed significant results (median, 53 months in the chemotherapy arm *vs.* 36 months in the observation group; *p*=0.028). Furthermore, a pre-specified intent-to-treat analysis adjusted for nodal status, disease grade and sex also showed significant difference between the two groups. In the present case, due to the aggressivity of the disease, combination of gemcitabine/capecitabine was chosen.

Since capecitabine cross the placenta and have been shown to be teratogenic and embryolethal, it was decided to start with gemcitabine during pregnancy and add capecitabine after delivery. Furthermore, due to low-risk emetic risk induced by gemcitabine, no prophylactic anti-emetic therapy was prescribed in this case.

Providing the maximum benefit to the mother through early delivery and aggressive chemotherapy against aggressive form of cancers has clear drawbacks related to the induction of premature delivery and possible side effects of chemotherapy on the fetus, such as growth restriction, and anemia secondary to bone marrow suppression. Currently, it is widely accepted that chemotherapy can be safely administered during the second and third trimesters, always with close monitoring of the mother and fetus. The blood placenta barrier limits fetal exposure to cytotoxic drugs with a different transplacental capacity for various drugs (11). Furthermore, placental dissemination, as a rare manifestation of cancer in pregnancy, comprises in the first place a direct infiltration of placental tissue and less probably the presence of malignant cells in the intervillous space corresponding to invasion in the maternal vascular compartment. Owing to the anatomical and immune barrier of the placenta, fetal metastases are even less common (12).

To our knowledge, there are no studies describing whether the human placenta is an effective barrier to gemcitabine but only case reports (**Table 1**). The treatment was aligned with the assessment done by Briggs (18), considering: (a) gemcitabine molecular weight and negligible plasma protein binding (b) animal data (6) (c) trimester of pregnancy (avoiding first trimester which represents organogenesis) (d) very short plasma elimination half-life reducing the amount reaching the embryo–fetus allowing gemcitabine use from the second trimester of pregnancy.

**Table 1:** Clinical experience with gemcitabine-based chemotherapy in pregnancy.

First author, year	Kim, 2008 (13)	Gurumurthy, 2009 (14)	Lubner, 2011 (15)	Wiesweg, 2014 (16)	Steinberg, 2020 (17)
Diagnosis	metastatic pulmonary adenocarcinoma + brain met	non-small-cell lung cancer	pancreatic adenocarcinoma	Metastatic cholangiocellular adenocarcinoma	Hodgkin lymphoma
Patient age [years]	35	38	37	38	29
Gestational age at treatment initiation [weeks]	9-22	25	24-31	18	Second trimester
Initial disease stage	stage IV with brain metastases	stage IV with bilateral lung metastases	Stage III		Relapse
Initial treatment intent	palliative	palliative	curative	palliative	Salvage therapy
Chemotherapy Doses et durées d’exposition	1st line: docetaxel and cisplatin (days 1 and 8) every 3 weeks) At 19 weeks, therapy was changed to gemcitabine and cisplatin (days 1 and 8) at 3- week intervals for two cycles 3rd line: gefitinib	gemcitabine (1000 mg/m2 on day 1 and 8) and carboplatin AUC 5 (day 1)	gemcitabine (1000 mg/m2) beginning her 24th week of pregnancy, until her 31st week.	Chimio semaine 18-29 cisplatin 50 mg/m2 on day 1 and gemcitabine 1,000 mg/m2 on days 1 and 8 of a 21-day cycle	gemcitabine, navelbine, doxorubicin liposome (3 cycles)
Response to gemcitabine-based treatment	progression	progression after 1 cycle		partial remission of the hepatic lesion. no signs of recurrent disease in the region of previous surgery. However, a slight progression of a previously very small pelvic metastasis was reported. Confirming our clinical impression during the previous weeks, bone metastases of the frontotemporal cranium, ribs, and ilium were revealed by bone scintigraphy and cranial CT. Abdominal MRI confirmed progressive disease in the liver and bone metastases with large soft tissue components.	progression
Outcome of the infant	About 2 months after the last dose, her pregnancy was diagnosed. A cesarean section was performed at 33 weeks to deliver a normal, 1490-g female infant (normal karyotype 46, XX) with Apgar scores of 8, 9, and 10 at 1, 5, and 10 minutes, respectively, and normal blood counts. An extensive examination of the infant failed to find any abnormalities. She was developing normally at 10 months of age	elective cesarean section at 28 4/7 weeks to a baby girl with Apgar scores of 7 and 9 at 1 and 5 minutes, respectively. The infant had multiple complications secondary to prematurity. The authors speculated that two unusual features, chronic lung disease and excessive secretions from her lungs, might have been related to either the chemotherapy or an effect of the malignancy itself. At 8 months of age, the infant had been weaned off of oxygen therapy and her neurodevelopment was age- appropriate	At 35 weeks, labor was induced to deliver a normal male infant with blood counts within normal limits. He was developing normally with a functionally intact immune system at nearly 2 years of age. The mother died 12 months after diagnosis (relapse was diagnosed 2 weeks after delivery, and 6 weeks off chemotherapy)	35 + 0, delivering a healthy female infant, APGAR 9/10/10, birth weight 1,840 g (20th percentile), length 41 cm (20th percentile), and head circumference 30 cm (30th percentile). The baby initially showed labored breathing with retractions but did not require supplemental oxygen, breathing spontaneously at all times. Examination revealed no evidence of gross malformations, neurological or cardiocirculatory abnormalities, and infection or bone marrow suppression (leukocytes 6.85/nL, hemoglobin 14.5 g/dL, and platelets 320/nL). Supplemental enteral feeding *via* nasogastric tube was necessary for 10 days. The baby was discharged from the hospital in very good general condition 1 month after her birth. Weight was 2,340 g (< third percentile), height 42 cm (< third percentile), and head circumference 31 cm (< third percentile).	healthy boy weighing 2.93 kg
Delivery time [weeks+days]	33	28+4		35	Third trimester
Follow-up patient	available during the first 10 months	the patient died 2 weeks postpartum		Although her head circumference was small, there were no neurological and behavioral defects and development was normal during 14 months of follow-up. A pediatric follow-up examination of the infant after a corrected age of 5 months confirmed normal physical and neurological development. In a second examination at a corrected age 12 months, development was found	Post-delivery PET-CT showed progression of disease, and palpable nodes had begun to grow again.
				again appropriate for her age, with length at 1 cm below 3rd percentile and weight at the 10th percentile. The neuropediatric examination showed no abnormal findings.	
				Chemotherapy with cisplatin and gemcitabine was resumed, palliative local radiotherapy administered to the sacrum, ilium, and femoral neck at a total dose of 20 Gy, and bisphosphonate therapy initiated.	
				Only 2 weeks after the completion of the radiotherapy, disease progressed with new vertebral, mediastinal, and pulmonary metastases. Thus, second-line chemotherapy with 5-FU, folic acid, and oxaliplatin was initiated.	
				Third-line therapy with paclitaxel was not tolerated due to severe polyneuropathy. Hence, weekly epirubicin was administered as fourth-line therapy. Fourteen months after the initial diagnosis massive progression of pulmonary, hepatic and bone metastases occurred. The patient died shortly afterward.	

Recently, a descriptive cohort study of 1,170 pregnant cancer patients (39% with breast cancer) aimed at analyzing changes over time in cancer, obstetric, and neonatal outcomes. The authors observed an increase in the use of chemotherapy during pregnancy, in parallel with an increase in live births, and a reduction in iatrogenic preterm deliveries, encouraging the management of these patients in high obstetric care units (19). Before starting any oncological treatment, a fetal examination with ultrasound should be performed after 8th week to exclude pre-existing abnormalities, because exposure to cytotoxic agents in the first trimester can interfere with fetal organogenesis, resulting in an increased risk of miscarriages and congenital malformations. If there is an urgent need to start chemotherapy during pregnancy, the option of preterm labor to avoid any delay in treatment initiation should be carefully discussed with the patient.

The present case represents the first description in the literature of gemcitabine administration in a pregnant woman with biliary tract cancer, successfully treated with surgery and adjuvant chemotherapy during gestation period, leading to a disease-free interval of more than 3 years. **Table 1** summarize other case reports describing the results of gemcitabine-based chemotherapy during pregnancy. In addition, no acute or late side effects of chemotherapy were observed in the infant during 3-year follow-up.

## Conclusion

A multidisciplinary approach involving surgeons, obstetricians, neonatologists and oncologists is required. In addition, shared decision-making with the patient and family members is indispensable in the management of cancer diagnosed during pregnancy. In addition, the maternal benefit of gemcitabine seems to outweigh the unknown fetal risk.

The present case report demonstrates that surgery of gallbladder cancer followed by adjuvant chemotherapy with gemcitabine can be carried out safely and effectively in the gestational period.

## Ethics statement

Written informed consent was obtained from the individual(s) for the publication of any potentially identifiable images or data included in this article.

## Author contributions

AD (11^th^ author), ND, PS contributed to the conception and design of the study. AD (1^st^ author) wrote the first draft of the manuscript. AM, PS, MG wrote sections of the manuscript. All authors contributed to manuscript revision and read and approved the submitted version.

## Funding

Open access funding was provided by the University of Lausanne.

## Conflict of interest

The authors declare that the research was conducted in the absence of any commercial or financial relationships that could be construed as a potential conflict of interest.

## Publisher’s note

All claims expressed in this article are solely those of the authors and do not necessarily represent those of their affiliated organizations, or those of the publisher, the editors and the reviewers. Any product that may be evaluated in this article, or claim that may be made by its manufacturer, is not guaranteed or endorsed by the publisher.
