# Erratum: Gallbladder cancer during pregnancy treated with surgery and adjuvant gemcitabine: A case report and review of the literature

**DOI:** 10.3389/fonc.2023.1175336

**Published:** 2023-03-16

**Authors:** Frontiers Production Office

**Affiliations:** Frontiers Media SA, Lausanne, Switzerland

**Keywords:** gallbladder cancer, chemotherapy, pregnancy, gemcitabin, biliary tract cancer

An omission to the funding section of the original article was made in error. The following sentence has been added: “Open access funding was provided by the University of Lausanne”.

The original version of this article has been updated.

